# Deletion of the Chemokine Binding Protein Gene from the Parapoxvirus Orf Virus Reduces Virulence and Pathogenesis in Sheep

**DOI:** 10.3389/fmicb.2017.00046

**Published:** 2017-01-24

**Authors:** Stephen B. Fleming, Catherine McCaughan, Zabeen Lateef, Amy Dunn, Lyn M. Wise, Nicola C. Real, Andrew A. Mercer

**Affiliations:** Department of Microbiology and Immunology, University of OtagoDunedin, New Zealand

**Keywords:** Orf virus, poxvirus, chemokine binding protein, virus immune modulation, pathogenesis, virulence

## Abstract

Orf virus (ORFV) is the type species of the *Parapoxvirus* genus of the family *Poxviridae* and infects sheep and goats, often around the mouth, resulting in acute pustular skin lesions. ORFV encodes several secreted immunomodulators including a broad-spectrum chemokine binding protein (CBP). Chemokines are a large family of secreted chemotactic proteins that activate and regulate inflammation induced leukocyte recruitment to sites of infection. In this study we investigated the role of CBP *in vivo* in the context of ORFV infection of sheep. The CBP gene was deleted from ORFV strain NZ7 and infections of sheep used to investigate the effect of CBP on pathogenesis. Animals were either infected with the wild type (*wt*) virus, CBP-knockout virus or revertant strains. Sheep were infected by scarification on the wool-less area of the hind legs at various doses of virus. The deletion of the CBP gene severely attenuated the virus, as only few papules formed when animals were infected with the CBP-knock-out virus at the highest dose (10^7^ p.f.u). In contrast, large pustular lesions formed on almost all animals infected with the *wt* and revertant strains at 10^7^ p.f.u. The lesions for the CBP-knock-out virus resolved approximately 5–6 days p.i, at a dose of 10^7^ pfu whereas in animals infected with the *wt* and revertants at this dose, lesions began to resolve at approximately 10 days p.i. Few pustules developed at the lowest dose of 10^3^ p.f.u. for all viruses. Immunohistochemistry of biopsy skin-tissue from pustules showed that the CBP-knockout virus replicated in all animals at the highest dose and was localized to the skin epithelium while haematoxylin and eosin staining showed histological features of the CBP-knockout virus typical of the parent virus with acanthosis, elongated rete ridges and orthokeratotic hyperkeratosis. MHC-II immunohistochemistry analysis for monocytes and dendritic cells showed greater staining within the papillary dermis of the CBP-knock-out virus compared with the revertant viruses, however this was not the case with the *wt* where staining was similar. Our results show that the CBP gene encodes a secreted immunodulator that has a critical role in virulence and pathogenesis.

## Introduction

Orf is a debilitating skin disease of sheep and goats and is transmissible to man (Fleming et al., 2015). Orf is also known as contagious ecthyma, pustular dermatitis, or scabby mouth and is caused by the parapoxvirus orf virus (ORFV) (Haig, 2006). Orf is an economic and welfare problem in sheep and goat producing countries world-wide (Haig, 2006). The disease around the mouth of lambs is associated with poor growth and can become fatal when secondary infections occur (Fleming et al., 2015). The disease is usually benign, with lesions that progress through a papular stage to form pustules within a few days. These rupture giving rise to ulcers and subsequently a thick and overlying crust that is shed within 4–6 weeks (Fleming and Mercer, 2007).

The vaccine against orf is live unattenuated virus that only produces short-term immunity (Haig and McInnes, 2002).

ORFV usually infects the host through breaks and abrasions to the skin and replicates in regenerating keratinocytes (McKeever et al., 1988). Keratinocytes constitute approximately 90% of the cells within the epidermis and are recognized as immune sentinels within skin (Debenedictis et al., 2001; Nestle et al., 2009). They express a wide range of Toll-like receptors and other sensory molecules that allow these cells to respond rapidly to infection by producing proinflammatory cytokines and chemokines that results in the recruitment of immune cells from the underlying dermis and blood during infection.

Genetic analyses have revealed a number of viral encoded factors that may explain its ability to establish infection within the specific immune environment of the skin and its ability to reinfect its host (Fleming et al., 2015). Several secreted immunomodulators have been described and these include a vascular endothelial growth factor (Lyttle et al., 1994; Wise et al., 1999; Savory et al., 2000), a factor that binds IL-2 and GM-CSF (Deane et al., 2000), an IL-10-like molecule (Fleming et al., 1997) and a chemokine binding protein (CBP) (Seet et al., 2003; Lateef et al., 2009, 2010; Counago et al., 2015).

Chemokines are a large family of secreted chemotactic proteins that activate and regulate inflammation induced leukocyte recruitment to sites of infection as well as homeostatic migration of leukocytes through lymphoid organs (Baggiolini, 1998; Cyster, 1999). Members of the family are classified as CC, CXC, CX_3_C, and C according to the spatial arrangement of cysteine residues within the N-terminus of the molecule. In general CC chemokines are chemoattractants for monocytes (Uguccioni et al., 1995) whereas CXC chemokines are chemoattractants for lymphocytes, NK cells, neutrophils and B cells (Huang et al., 2001). Chemokines are highly conserved across mammalian species. Leukocytes are recruited to sites of inflammation by interaction with chemokine gradients that form by attachment to the extracellular matrix through glycosaminoglycan binding domains (Yu et al., 2005). The predominant chemokines produced during damage and inflammation of epithelial tissues are CCL2 (MCP-1), CCL3 (MIP-1a), and CCL5 (RANTES) (Kopydlowski et al., 1999; Wetzler et al., 2000).

Previous studies have revealed that the ORFV CBP is related to the poxvirus type II CC-chemokine binding proteins (CBP-II) produced by the *Orthopoxvirus* and *Leporipoxvirus* genera (Seet et al., 2003). Although ORFV CBP displays low sequence identity to other poxvirus CBPs (<17%) it shares regions of identity and similarity across its entire sequence. The ORFV CBPencoded by strain NZ2 has been extensively characterized and is functionally similar to the CBP-II proteins in its ability to bind many human inflammatory and constitutive CC-chemokines with high affinity but also binds XCL1 (lymphotactin) and several CXCL chemokines (Seet et al., 2003; Lateef et al., 2009, 2010; Counago et al., 2015). However, the significance of ORFV CBP during infection of its natural host has yet to be examined.

We hypothesized that the ORFV CBP disrupts chemokine gradients within the skin tissue of its host during infection and thereby sets up a blockade to inhibit the recruitment of leukocytes to the infected site. For these studies we used ORFV strain NZ7. The NZ7 strain induces large pustular lesions in sheep and has been used to characterize other ORFV virulence factors (Fleming et al., 2007; Wise et al., 2007). We firstly characterized the chemokine binding properties of the purified CBP protein encoded by ORFV strain NZ7 (CBP_NZ7_) by ELISA and its effects on monocyte migration in response to specific chemokines in a chemotaxis assay and inflammatory cell trafficking using a murine skin inflammation model. To examine the *in vivo* effects of this factor on pathogenesis and host response we constructed a knock-out recombinant in which the CBP gene was deleted. Infection studies were carried out in sheep using the recombinant virus and the clinical pathology and host response compared with the parent strain.

## Materials and methods

### Virus and cells

The ORFV_NZ7_ strain (Robinson et al., 1987) was propagated in primary lamb testis (LT) cells as described previously (Robinson et al., 1982). LT cells were maintained in minimum essential medium (MEM) (GIBCO, Invitrogen) and supplemented with FBS at 10% for growth and 5% for culture maintenance. LT cells were supplemented with PKS solution [kanamycin, (Roche Life Science); streptomycin. penicillin (Gibco)]. Cells were incubated at 37°C in a humidified 7% CO_2_ atmosphere.

### Expression and purification of CBP

The accession numbers for ORFV strain NZ2 CBP and ORFV strain NZ7 CBP are CAD99366 and AY453066 respectively. The coding region of the CBP gene from ORFV strain NZ7 was amplified by PCR from the Hind-III-E fragment (Robinson et al., 1987). The primers used for amplification were N.term 5′-cgcggatccgccaccatgaaggcggtgttgttgct and C.term 5′-cgcggatccttacttgtcatcgtcgtccttgtagtcatggccagggttgaggttaa and were based on the 5′ and 3′ ends of the CBP coding region, respectively. Each primer possessed a BamHI site to allow cloning into the plasmid pAPEX-3 (a gift from Clare McFarlane, Walter and Eliza Hall, Institute, Melbourne). This cloning step incorporated a Kozak sequence and FLAG sequence at the 5′ and 3′ ends respectively, of the coding sequence. The plasmid pAPEX-3 contains a simian virus 40 promoter sequence, transcription termination sequence, and a gene for hygromycin resistance. The protein was expressed in HEK293 cells and purified by affinity chromatography with M2 affinity gel by methods described previously (Seet et al., 2003; Lateef et al., 2009).

### Binding analysis of CBP activity by ELISA

Chemokine binding activity was measured indirectly by the ability of the CBP protein to interfere with the detection of the chemokines by specific ELISA (Deane et al., 2009; Counago et al., 2015; Lee et al., 2015). CCL2 OPTI EIA ELISA kits (BD Biosciences, Franklin Lakes, NJ, and CCL3, CCL5, CCL19, and CXCL2 DuoSet ELISA Development kits (R&D Systems Inc, Minneapolis, MN) were used in accordance with the manufacturer's instructions. Detailed methods for the ELISA have been described recently (Lee et al., 2015). Briefly MaxiSorp 96-well immunoplates (Nalgene Nunc) were coated with capture antibody. In non-absorbent 96-well plates murine chemokines CCL2, CCL3, CCL5, CCL19, and CXCL2 were mixed with increasing amounts of CBP diluted in PBS (Lee et al., 2015). The chemokine/CBP mixes were incubated for 45 min at 37°C and then transferred with chemokine standards to the coated plates for a further 15 min incubation at 37°C. The plates were washed with PBS/0.005% Tween-20, then incubated for 1 h at room temperature with biotinylated detection antibody and streptavidin-conjugated horseradish-peroxidase. After a final wash, captured chemokine was detected with 3,3′,5,5′-tetramethylbenzidine (BD Biosciences), and the absorbance measured at 450 nm.

### Murine skin inflammation model

The mouse experiments described in this study were approved by the University of Otago Animal Ethics Committee. Specific Pathogen Free C57BL/6 mice and enhanced green fluorescent protein (eGFP) transgenic C57BL/6 mice were obtained from the University of Otago Animal Facility and used with institutional ethical approval. Monocyte and DC recruitment from the blood was assessed using adoptive transfer of eGFP bone marrow transgenic cells into sex-matched 6–8 week old C57BL/6 recipients as described previously (Lateef et al., 2009, 2010). Bone marrow cells were collected from eGFP transgenic C57BL/6 donor mice and resuspended in DPBS (without FCS), and 3 × 10^7^ cells were administered to each recipient in a 200 ul volume in the tail vein. Twenty-four hours later, inflammation was induced in the skin by lipopolysaccharide [LPS, (*Eschericia coli* derived, Sigma)] intradermal injection. The optimal levels of LPS to inject into the dermis of the shaved abdomen of C57BL/6 mice and the time at which maximal monocyte and DC recruitment was evident, has been described previously (Lateef et al., 2009, 2010). Briefly, Evans blue dye (2 μl of 1% solution) was mixed in each sample (total volume 20 μl) to visualize the rate of diffusion of injected material. A day later animals were euthanized and the skin around the intradermal injection site was excised and weighed prior to incubating in collagenase dispase (Roche Diagnostics) for 3 h. The isolated cells were counted using a haemocytometer and stained for cell surface markers CD11b (allophycocyanin-conjugated rat anti-mouse integrin/CD11b, clone M1/70, isotype IgG_2b_; BD Biosciences) and Gr-1 (anti-Gr-1 allophycocyanin-conjugated, clone RB6-8C5, isotype IgG2b; R&D Systems) for monocytes or CD11c (allophycocyanin-conjugated clone HL3, isotype IgG1; BD Pharmingen) and MHC-II (rat anti-mouse I-A/I-E PE-conjugated from clone M5 (BD Pharmingen) at 4°C on ice for 2 h. Isotype control was IgG2b) for DC. Flow cytometric analysis was performed using a FACSCalibur (Becton Dickinson). All flow cytometric data were analyzed using CellQuest and FlowJo softwares. All samples were analyzed using flow cytometry counts of 10,000 events and were normalized to haemocytometer counts and weight of individual skin biopsies.

### Construction of recombinant ORFV

The CBP knock-out ORFV was made by methods described previously (Savory et al., 2000; Fleming et al., 2007). The CBP gene is located within the restriction endonuclease fragment Hind-III-E of ORFV_NZ7_ (Seet et al., 2003) GenBank accession No AY453066. A fragment that spans 500 bp immediately upstream of the CBP coding region (left arm), was PCR amplified and cloned into the Hind-III and EcoRI restriction sites of pSP70 to generate pSP70/left arm. A fragment which spans 556 bp downstream of the CBP coding region and which includes 18 bp of the CBP gene (right arm), was PCR amplified and cloned into the vector pSP70/left arm at the EcoRI and BgI-II restriction sites. Finally the *Eschericia coli gene* (lacZ), placed under the control of an ORFV late promoter PF1 (Fleming et al., 1993), was cloned as a reporter gene into an EcoRI site located between the left and right arms. The construct was designed to delete 870 nts from the coding sequence of the CBP gene and to replace this sequence with ß-galactosidase. The final plasmid construct was called pCBPnz7-Δ. The recombinant CBP knock-out (CBP-ko) virus was generated using a procedure adapted from standard protocols used in the generation of vaccinia virus recombinants (Mackett et al., 1984) and methods described previously (Savory et al., 2000). Putative recombinant plaques were identified 4–5 days after infection by their blue plaque phenotype in the presence of 5-bromo-4-chloro-3-indoyl B-D galactopyranoside (X-Gal).

To construct the CBP revertant, a 1398 bp fragment spanning 500 bp upstream of the CBP coding sequence and which included the CBP coding sequence and transcription termination sequence T5NT, was PCR amplified and cloned into the Hind-III and Kpn-I restriction sites of pSP70 (left arm). In a further construct to produce a CBP-FLAG fusion protein, the FLAG sequence was incorporated at the C-terminus of the CBP coding sequence, that is a FLAG sequence was also incorporated into a second primer to make the left arm by amplification as described above. A fragment which spans 556 bp downstream of the CBP coding region and which includes 18 bp of the CBP gene (right arm used to make the knock-out construct), was cloned into the vector pSP70/left arm at the EcoRI and BgI-II restriction sites. The reporter gene ß-glucuronidase (GUS) running off an early/late promoter was then inserted into the KpnI and EcoRI site, that is between the left and right arms of the construct. This placed the GUS gene downstream of CBP in an intergenic region in the ORFV-CBP revertant. In addition an early transcription termination sequence, TTTTTCT, was engineered immediately downstream of the GUS gene. The final plasmid construct was called pCBP-rev. The recombinant virus was generated from the ORFV CBP-ko recombinant by methods described above. Putative ORFV-CBP revertant plaques were identified by their blue plaque phenotype in the presence of 5-bromo-4-chloro-3-indolyl B-D-glucuronide cyclohexylammonium salt and lack of blue color in the presence of X-gal. The revertant virus was named CBP-rev. A second revertant was made that incorporated a FLAG sequence at the C-terminus of CBP and named CBP_FLAG_-rev. Characterization of the genomic DNA isolated from the recombinants was carried out by restriction endonuclease and PCR analysis by methods described previously (Tan et al., 2009).

### Northern blotting

To analyse mRNA from virus infected cells by Northern blotting, total RNA was isolated by methods described previously (Fleming et al., 1991, 1993). Briefly lamb testis cells (LT), pretreated with cycloheximide (100 μg/ml), were infected with virus at an MOI of 30 pfu/cell and incubated for 6 h. Total RNA was isolated from cells using an RNeasy (Quiagen) purification kit following the manufacture's instructions. RNA was stored in 2.5 vol. ethanol, 0.05 vol. 5M NaCl at −70°C. RNAs were electrophoresed in denaturing agarose-formaldehyle gels, blotted onto nitrocellulose membranes and hybridized with a nick-translated CBP-specific ^32^P-labeled dsDNA probe. The size of mRNA was determined using a BRL RNA ladder. Autoradiography was performed for 72 h at −70°C.

### SDS-PAGE and immunoblotting

Affinity purified CBP-FLAG from LT cells infected with recombinant virus was diluted in SDS-PAGE sample buffer containing 5% 2-mercaptoethanol, boiled and then resolved by SDS-PAGE (Laemmli, 1970). Proteins were transferred to nitrocellulose and reacted with anti-FLAG Bio-M2 antibody as described previously (Achen et al., 1998). Blots were developed using a Super Signal West Pico Chemiluminescence (Perbio) ECL kit (Perbio).

### Infection of sheep

All animal experiments described in this study were approved by the University of Otago Animal Ethics Committee. The animals were housed in a PC2 containment facility at AgResearch, Invermay, Otago. Twenty-four ORFV sero-negative merino-cross lambs (ewes) were randomly divided into four groups of six and inoculated on the wool-free region of the inner hind legs. Group 1 was infected with *wt* virus, group 2 with CBP-ko, group 3 with CBP-rev virus and group 4 with the CBP_FLAG_- rev virus. Four lines of scarification approximately 5 cm in length were made on the epidermis of each of the hind legs and infected by topical application of 100 μl of PBS containing 10^7^, 10^5^ and 10^3^ p.f.u virus or PBS only (no virus control). Virus-induced lesions were photographed daily over a 3 week period and the clinical scores determined. Sheep were examined for erythema, papule and pustule formation and firmly attached scab associated with the scarified/infected areas of the skin. The clinical score was determined by counting the discrete round raised papules with a creamy appearance that formed on the skin surface along the lines of scarification using magnified photographic images and the width of the lesion (1–10 mm) that formed when papules coalesced (days 5–7). Clinical scores ranged from 1 to 25. Once the papules/lesions lost their creamy appearance, that is were in the process of forming scab, the clinical score was recorded as zero. The clinical scores for all animals at all-time points were scored by two researches. Punch biopsies (5 mm) were taken at days 4, 6, and 8 from the right leg. Blood was obtained from the jugular vein of each animal before infection and then at 13 and 18 days pi when all animals were euthanized.

### Detection of anti-ORFV antibodies by ELISA

Antibodies produced against ORFV antigen and ORFV-075 scaffold protein were analyzed in sheep serum by ELISA. ORFV antigen was prepared from ORFV-infected LT cells as described previously (Tan et al., 2012). The expression and purification of the ORFV-075 protein has been described previously (Hyun et al., 2007). Detailed methods describing the detection of anti-ORFV antibodies from sheep serum have been described (Bowden et al., 2009; Tan et al., 2012). Anti-ORFV antibodies were detected by coating 96-well Maxisorp Nunc imuno plates (Nunc) with 0.5 ug ORFV antigen per well and ORFV-075 with 10 ug/well. For pre-infection control (day 0), serum samples were assayed against specific antigen and no antigen and for post-infection control, samples were assayed against no antigen. Following overnight incubation at R/T washing and blocking with 5% (w/v) skimmed milk in PBS-T, 100 μl of sera were applied to the wells. Sheep sera were diluted 1/400. The plates were incubated for 1.5 h at R/T followed by 3 washes with PBS-T. Serum antibodies were detected using 100 μl of 1:2000 rabbit anti-sheep Ig-HRP. The plates were incubated for 1.5 h at R/T and washed in PBS-T prior to detection. One hundred microliter of o-phenylenediamine dihydrochloride (OPD) substrate solution (Sigma) was applied to all wells and color allowed to develop in the dark. The reactions were read at OD_490_ nm.

### Sheep skin histology: haematoxylin and eosin staining and immunofluorescence

Skin biopsies (5 mm) from sheep were treated with zinc salt fixative (González et al., 2001) and embedded in paraffin wax for cellular analysis. Sections were cut at 4 μm, mounted on glass slides and then dried at 60°C for 1 h. Sections were then re-fixed in zinc salts for 7 days at room temperature before bench drying. Adjacent sections were stained with either haematoxylin and eosin (H & E), or anti-MHC II.

#### Haematoxylin and eosin staining

H & E staining has been described previously (Anderson et al., 2001).

#### MHC-II by immunofluorescence

For immunofluorescent staining of MHC-II, slides were dried O/N at 37°C tissue sections were de-paraffinated and rehydrated with xylene for 15 min, 100% ethanol for 30 s, 100% 1-propanol for 30 sec, 90% 1-propanol for 30 s, and 70% 1-propanol for 30 sec. To rehydrate, sections were soaked in 0.85% NaCl for 5 min, followed by TBS for 30 min. For antigen retrieval, slides were immersed in 37°C TBS for 20 min, then room temperature TBS for 20 min. Sections were blocked in 10% sheep serum/TBS for 30 min at room temperature in a humidified box. Sections were then incubated with 0.02 mg/ml mouse anti-ovine MHC Class II:FITC (Serotec, MCA2228F) in 10 mg/ml BSA/0.05% Triton X/TBS, and incubated O/N at 4°C. DAPI 1 was added for 30 min before sections were washed three times in 0.05% Tween20 TBS, then rinsed in TBS only. Sections were mounted with SlowFade Gold Antifade Mountant (Invitrogen S36936). The stained sections were viewed by fluorescent microscopy using an Olympus BX51 and then photographed using an Olympus digital camera. Cell counts were performed using Image J Fiji software by subtracting background, increasing contrast, creating a RGB stack, selecting either the epidermis, or the papillary dermis, determined by the smooth collagen and elastic fibers then measuring percentage area that stained positive in the green field.

#### Detection of ORFV antigen by immunofluorescence

For immunohistochemical staining of the ORFV F1L envelope protein, tissue sections were de-paraffinated and rehydrated as described above. Sections were then blocked in TBS + 5% FCS for 30 min and reacted with anti-ORFV F1L monoclonal antibody (8D7) (Housawi et al., 1998; a gift from Peter Nettleton, Moredun Research Institute UK) diluted 1/2000 in 1% TBS /BSA/0.01% tritonX and incubated O/N at 4°C. Sections were then washed in 1% TBS and incubated with goat anti-mouse alexa 594 (1/400 dilution) and DAPI (1:200 dilution) for 1.5 h in the dark. The stained sections were viewed by light microscopy or fluorescent microscopy.

### Statistical analysis

The mouse skin inflammation data was analyzed by paired Student's *t*-test and the statistical analyses were performed using the MegaStat suite under Excel. The sheep clinical score data for ORFV lesions was analyzed using the Mann-Whitney test (http://math.usask.ca/~laverty/S245/Tables/wmw.pdf). The sheep MHC-II immunofluorescence data was analyzed by One-Way ANOVA with means comparison using Sidak's test (GraphPad Prism 6).

## Results

### CBP_NZ7_ shows broad-spectrum chemokine binding specificity

**Figure 1:**
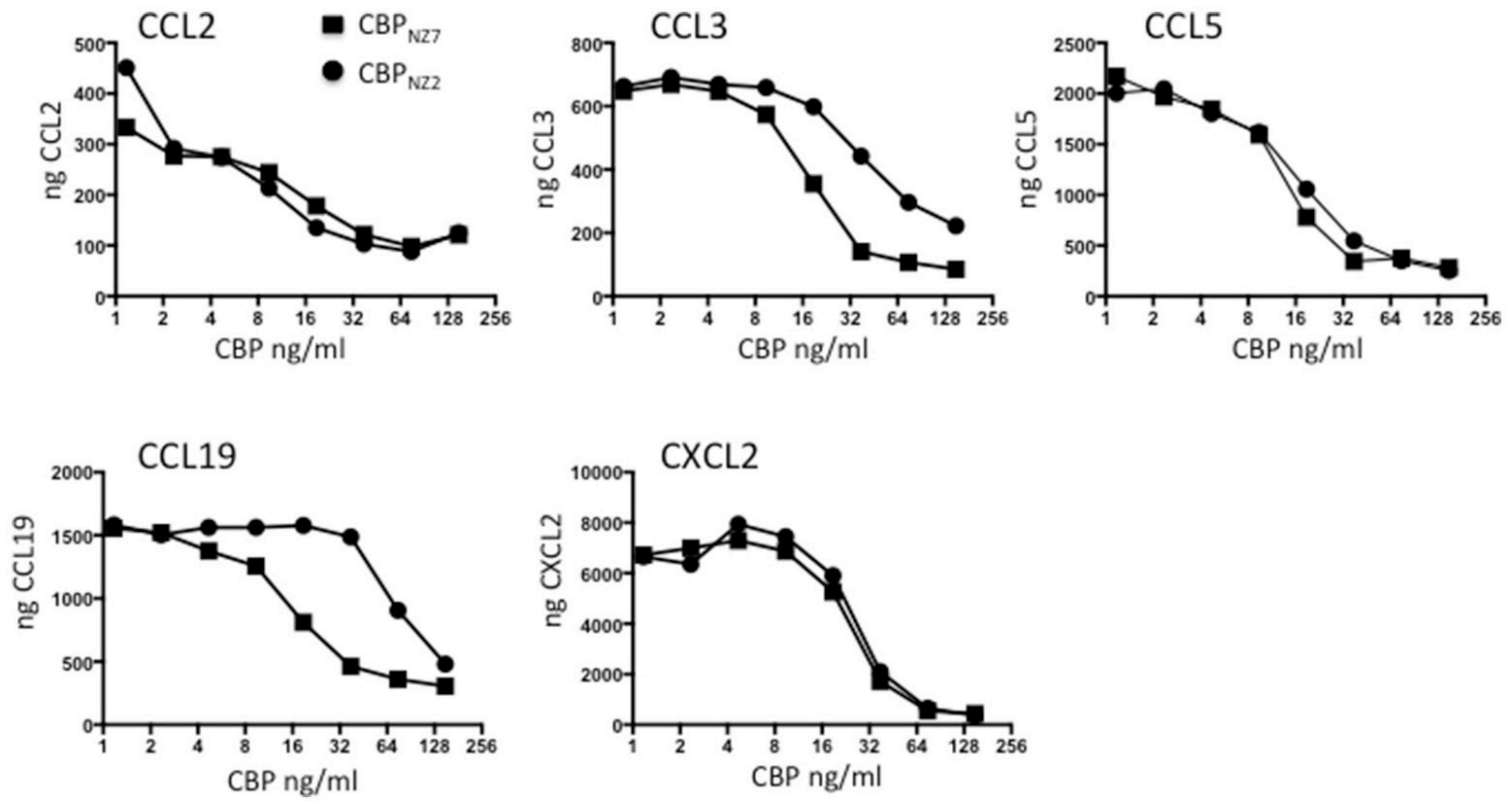
**CBP_**NZ7**_ is a broad-spectrum chemokine binding protein**. The chemokines shown were incubated with increasing amounts of either chemokine binding protein ORFV strain NZ7 (CBP_NZ7_) or CBP_NZ2_. Unbound chemokine was then detected using a capture ELISA. The results are presented as the total chemokine detected. High levels of chemokine indicate no interference in the detection of the chemokine (no chemokine binding) where as low levels of chemokine indicates interference with detection (positive chemokine binding).

The CBP gene shows surprising variability within the ORFV species with the strain NZ7 CBP showing only 78% identity to the strain NZ2 CBP at the amino acid level (Seet et al., 2003). These differences are partly due to three insertions in *CBP*_NZ7_ making the gene 27 nts longer than *CBP*_NZ2_ with a predicted polypeptide of 32.193 kDa compared with 31.182 kDa. It was therefore necessary to first examine the *in vitro* properties of CBP_NZ7_ and compare these with the published data of CBP_NZ2_. To examine the chemokine binding properties of CBP_NZ7_, the CBP_NZ7_ gene was cloned into the pAPEX eukaryotic expression vector and expressed as a C-terminal FLAG tagged fusion protein following transfection of HEK293 cells. The protein was affinity purified using anti-FLAG beads and tested for its ability to bind murine chemokines using an ELISA assay. The chemokine binding specificity of CBP_NZ7_ was compared with CBP_NZ2_ over a broad range of chemokines that are produced during inflammation in skin and the constitutive chemokine CCL19. The ELISA carried out is an indirect assay and measures free chemokine (not in complex with CBP) following CBP incubation with chemokine. Therefore, the stronger the binding the less free chemokine that remains. The assay showed that CBP_NZ7_ bound the murine inflammatory chemokines CCL2, CCL3, CCL5, and CXCL2 (Figure 1). It also bound the murine constitutive chemokine CCL19. Overall its binding specificities were similar to CBP_NZ2_ over the range of chemokines tested with stronger binding to CCL3 and CCL19, that is, there was less free chemokine available at a concentration of 37.5 ng/ml for CBP_NZ7_.

### CBP_NZ7_ inhibits inflammatory cell recruitment in a mouse skin inflammation model

**Figure 2:**
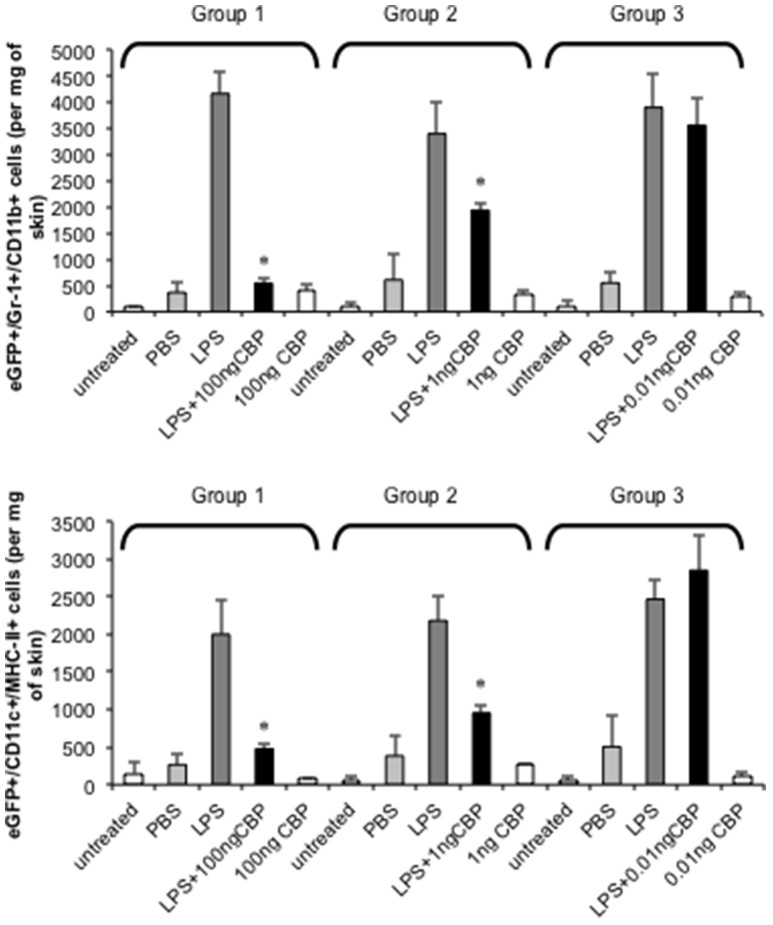
**CBP_**NZ7**_ inhibits LPS-induced recruitment of inflammatory monocytes and dendritic cells (DC) into skin**. Bone marrow cells from eGFP donor mice were transfused intravenously into recipient mice 24 h before intradermal injection of LPS (1 μg per site) with and without CBP_NZ7_. In addition each mouse received injections of CBP only and PBS for a total of four injections per mouse (each mouse within the group received LPS only, LPS + CBP, CBP only, PBS only and *n* = 3 animals per group for one experiment). Twenty-four hour later, the recruitment of eGFP/Gr1^+^/CD11b^+^ cells and eGFP/CD11c^+^/MHCII^+^ cells into the skin were analyzed. The bar represents the mean ± SD of 3 combined experiments (biological replicates) where *n* = 9 mice. Asterisks indicate results that are significantly different (*p* < 0.01; paired Students *t*-test).

The injection of small amounts of LPS in mouse skin results in highly localized inflammation through the upregulation of inflammatory chemokines that results in the recruitment of monocytes and DC to the site (Haberstroh et al., 2002; Charo and Ransohoff, 2006). The ability of CBP_NZ7_ to inhibit monocyte and DC recruitment to inflamed skin was tested. Optimization of the mouse skin inflammation model and analysis of the inflammatory cell infiltrate has been described previously (Lateef et al., 2009, 2010). We tested whether CBP_NZ7_ could impair recruitment of blood-derived monocytes and DC to the skin. Bone marrow cells from eGFP transgenic C57BL/6 donor mice were administered to C57BL/6 recipient mice. Twenty-four hours later animals were co-injected with 1 μg LPS with or without various amounts of CBP_NZ7_ (total volume 20 ul) into the dermis of the shaved abdomen of the recipients. Twenty-four hours later mice were euthanized and the skin around the injection site excised and cells isolated and stained. The number of monocytes and DC in skin was determined by flow cytometric analysis. The results showed that CBP_NZ7_ potently inhibited the recruitment of blood derived eGFP monocytes and eGFP DC at 1 and 100 ng of CBP_NZ7_ (*P* < 0.01, Students *t*-test for paired samples) with the greatest effect seen at 100 ng CBP_NZ7_ (Figure 2).

### Replication of the CBP-knock-out virus and revertants in cell culture and expression of CBP from the CBP_FLAG_-revertant virus

In order to study the effects of the ORFV CBP gene in the context of a viral infection in its natural a host, a CBP knock-out recombinant virus was made by homologous recombination. To construct the revertant viruses, the CBP gene was reinserted into the CBP knock-out virus. The growth of the recombinant viruses was compared with the parent virus in LT cells at 30 h post infection. All viruses exhibited similar growth and importantly the deletion of the CBP gene did not compromise its ability to replicate in cell culture (Figure S1). In addition transcription of CBP mRNA of the revertant viruses and the knock-out virus were compared with the parent virus by Northern blotting. The results showed that CBP mRNA could be detected in LT cells infected with the *wt* virus and the CBP revertant recombinants but not from the CBP knock-out virus (Figure S2). Expression of CBP-FLAG from the ORFV-CBP-FLAG-revertant was also examined by western blotting of virus-infected cells and the results showed that expression of CBP-FLAG could be detected for this recombinant (Figure S3).

### Infection of sheep with CBP knock-out ORFV showed reduced pathogenesis

**Figure 3:**
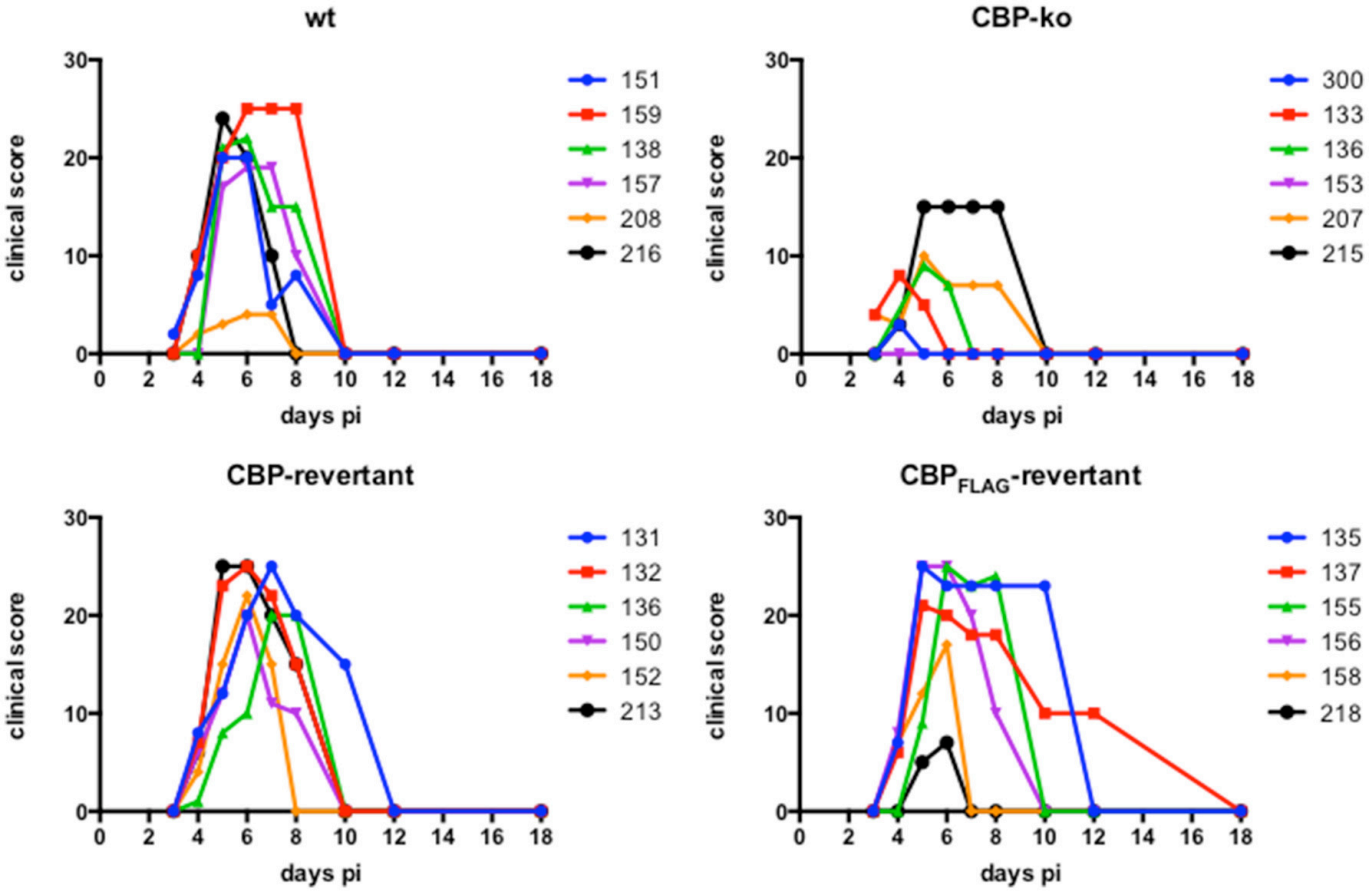
**ORFV lesion clinical scores of sheep infected with ***wt*** and recombinant viruses**. Infection with a dose of 10^7^ p.f.u. virus of each *wt*, CBP knock-out virus (ko) CBP revertant virus and CBP_FLAG_ revertant virus. There were 6 animals per group and the clinical scores are shown for each animal from day 3 to day 18 p.i. at which time the lesions in all animals had resolved (detached scab).

**Figure 4:**
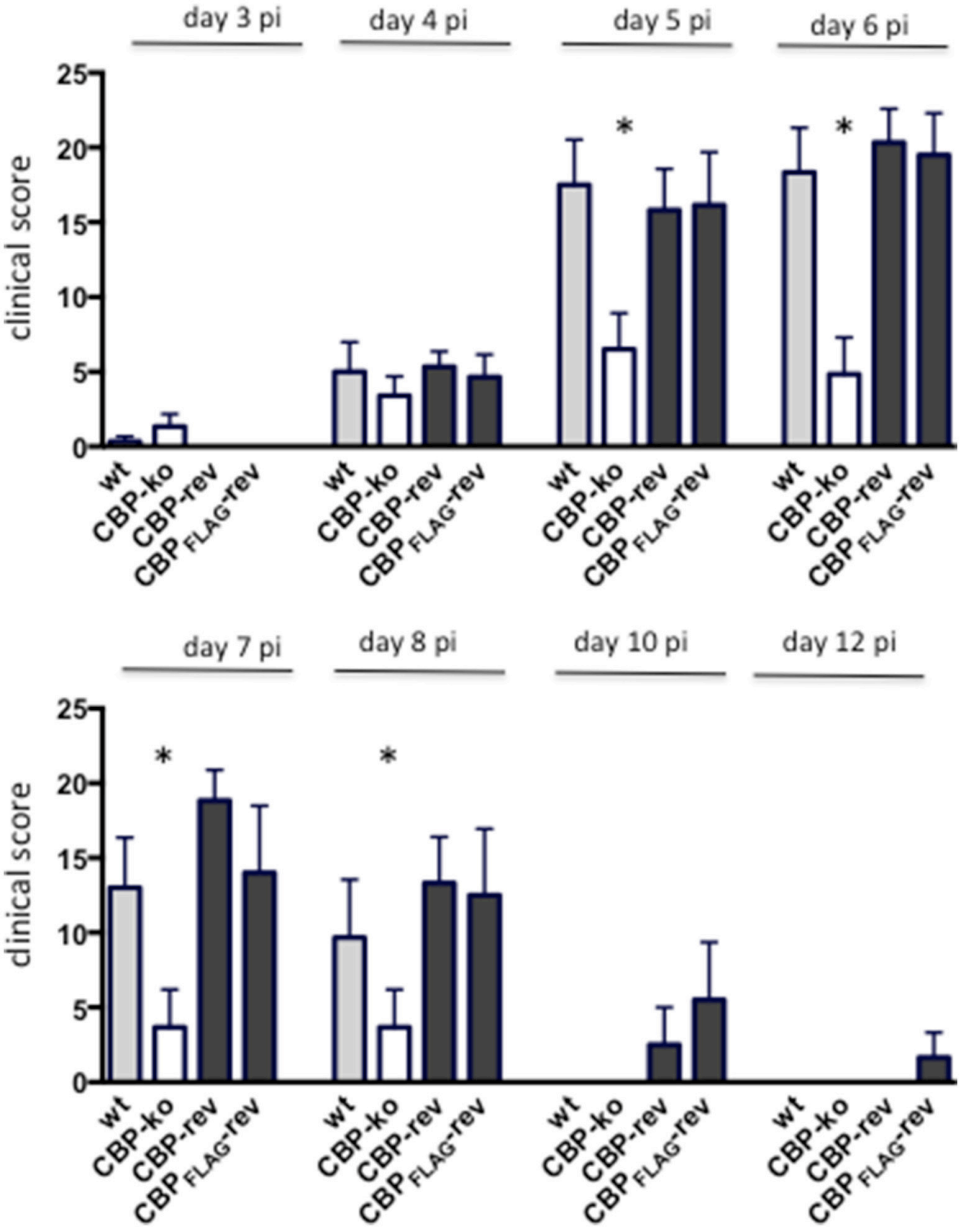
**The mean ORFV lesion clinical scores of sheep infected with ***wt*** and recombinant viruses**. The mean clinical score for each group infected with a dose of 10^7^ p.f.u. is shown. Asterisks indicate where the CBP-ko virus is significantly different to *wt* and revertants (*p* < 0.05, Mann-Whitney test).

**Figure 5:**
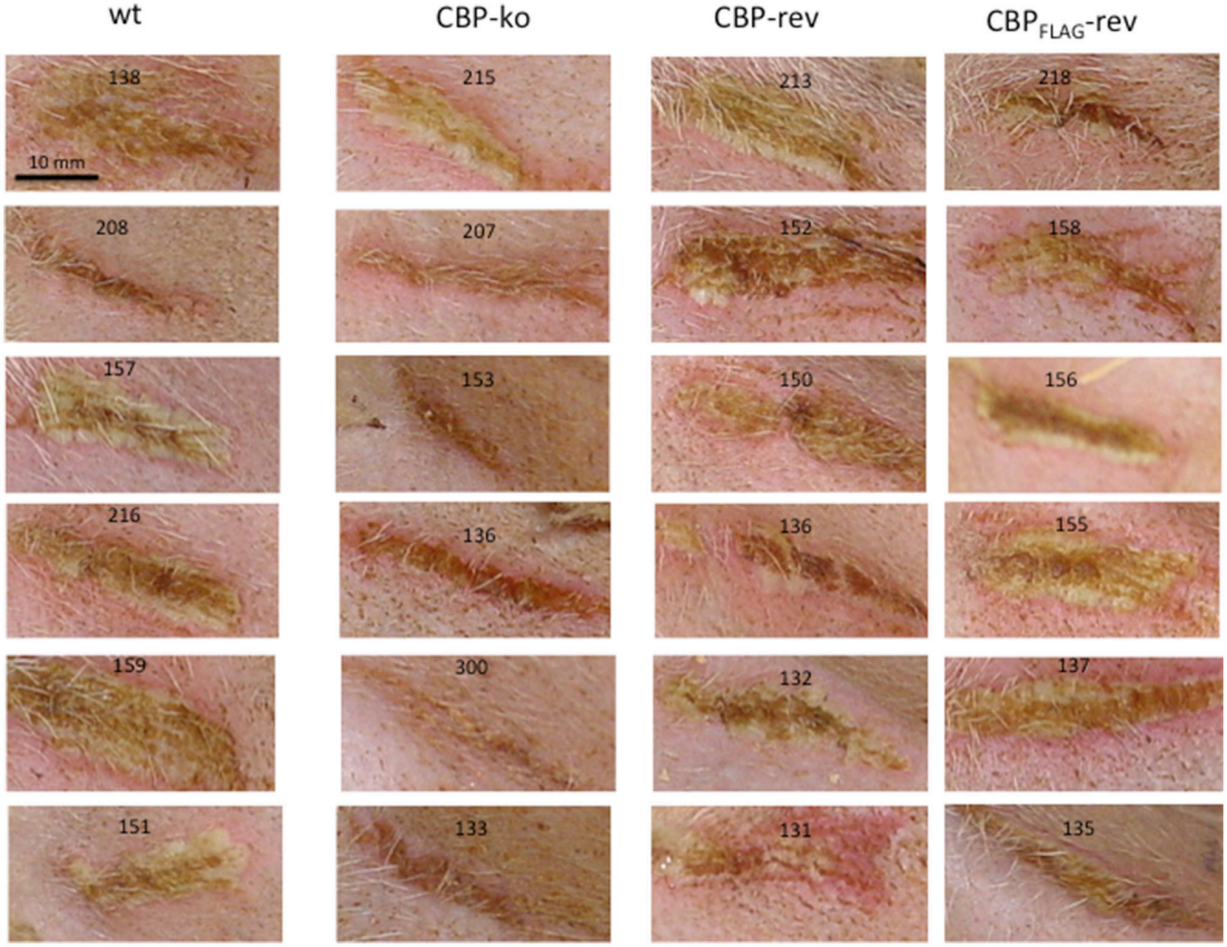
**Lesions of ORFV infected sheep at day 6 p.i., dose 10^**7**^ p.f.u. virus**.

Experimental infection of sheep with *wt* ORFV_NZ7_ by scarification of the skin generally results in discrete papules and pustules that form along the scratch line. As the lesions develop the pustules coalesce to form a continuous pustular lesion. In this study, groups of six animals were infected with either *wt*, CBP ko virus, CBP-rev virus or the CBP_FLAG_-rev virus at 3 doses; 10^7^, 10^5^, and 10^3^ p.f.u. virus and PBS (mock infected control). Infected skin was photographed to provide a record of the clinical pathology from which the clinical score could be determined from the number, appearance and size of papules and pustules that developed along the scratch line. The clinical score for each animal infected with a dose of 10^7^ pfu virus is shown in Figure 3 over the course of the infection and the mean clinical scores for each group during the course of infection is shown in Figure 4. By day 3 p.i., 2 animals infected with the CBP ko virus showed early development of papules and pustules that were not seen at this time in animals infected with either the *wt* virus or CBP revertant viruses. By day 4 p.i., 4 of the animals in the ko group had developed small pustules. Sheep infected with *wt* and the CBP revertants developed lesions typically observed for the NZ7 strain of ORFV and papules and pustules were visible by day 4 p.i. By day 5 p.i., large pustular lesions had developed in the *wt* and CBP revertant groups and the clinical scores were significantly greater than sheep infected with the CBP ko virus (*P* < 0.05). In contrast, lesions in the CBP knockout group were beginning to resolve by day 5 p.i. By day 6 p.i., lesions in the *wt* and CBP revertants groups had reached their maximum clinical pathology (Figures 3–5) and there was little difference in the mean clinical scores at this time; *wt* 18.33 (SE 2.99), CBP rev 20.33 (SE 2.26), CBP_FLAG_ rev 19.50 (SE 2.80). In contrast, lesions had resolved in 3 of the animals in the CBP knock-out group by this time and the group had a mean clinical score of 4.83 (SE 2.46). The clinical scores for the *wt* and CBP revertant groups were significantly different to the CBP knockout group (*P* < 0.02) at day 6 p.i. By day 7 p.i., lesions had resolved in all but 2 animals in the CBP knock-out group. By day 8 p.i. the clinical pathology in almost all animals was less apparent and lesions were beginning to form scabs. By day 10 p.i., scabs had formed on all animals except for 3 in the CBP revertant group. By day 18 p.i., lesions had resolved on all animals and scab had detached. Throughout the course of the experiment, the clinical scores for the *wt* and revertant groups were not significantly different to each other.

**Figure 6:**
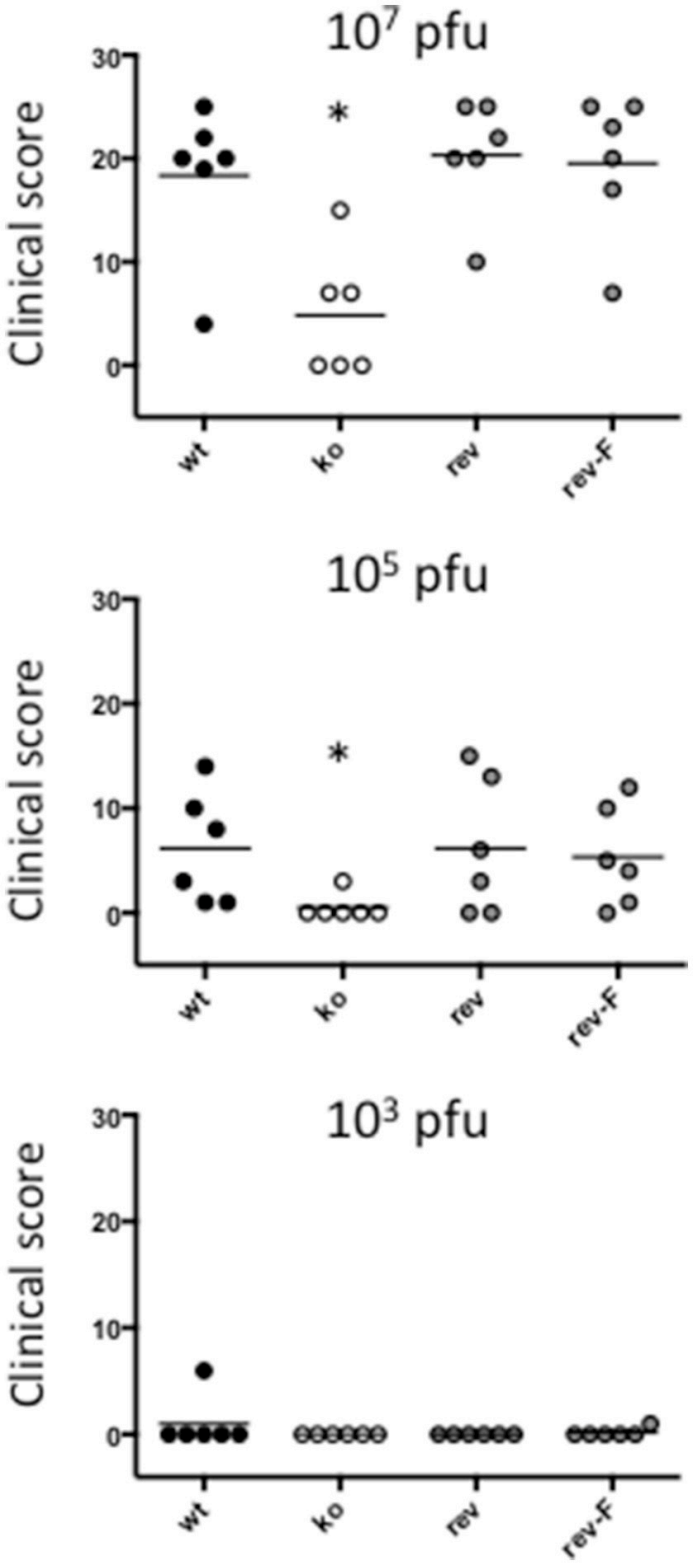
**ORFV lesion clinical scores of sheep from infection with three different doses of virus (10^**3**^, 10^**5**^, and a 10^**7**^ p.f.u.) of ***wt***, CBP-ko, CBP-revertant and CBP_**FLAG**_- revertant at day 6 p.i**. There were 6 animals per group and the clinical scores are shown for each animal. The bar represents the mean clinical score for each group and asterisks indicate where the CBP-ko virus is significantly different to *wt* and revertants (*p* < 0.05, Mann-Whitney test).

At an infectious dose of 10^5^ virus per inoculum, the trend in clinical pathology at day 6 was similar to that for animals infected with a virus dose of 10^7^ (Figure 6). The mean clinical scores for the *wt* group was 6.12 (SE 2.18) and for the CBP rev and CBP_FLAG_ rev groups 4.50 (SE 2.29) and 5.33 (SE 1.96) respectively. In contrast the clinical score for the CBP ko virus group was 0.50 (SE 0.50). The clinical scores for the *wt* and the CBP revertant groups were significantly different to the CBP knock-out group (*P* < 0.05). At 10^5^ virus per inoculum the lesions resolved in less time than for the 10^7^ dose administered. At a dose of 10^3^ virus per inoculum few animals developed lesions and these were only seen in the *wt* and revertant groups (Figure 6).

In summary the deletion of the CBP gene had a marked effect on the clinical pathology. Within the first 3 days many typical papules formed in the CBP ko group at the highest dose, however, surprisingly few of these papules progressed further to form pustules and in fact diminished with time. There was only one sheep infected with a CBP deletion mutant that showed signs of pustular development by day 6 p.i. In contrast all animals infected with either *wt* virus or the CBP revertants developed pustules by this time. In many animals the number and size of pustules infected with the revertants were comparable with the *wt* and resolved in approximately the same time.

### Immunohistochemical analysis showed that the CBP knock-out virus replicated in sheep

**Figure 7:**
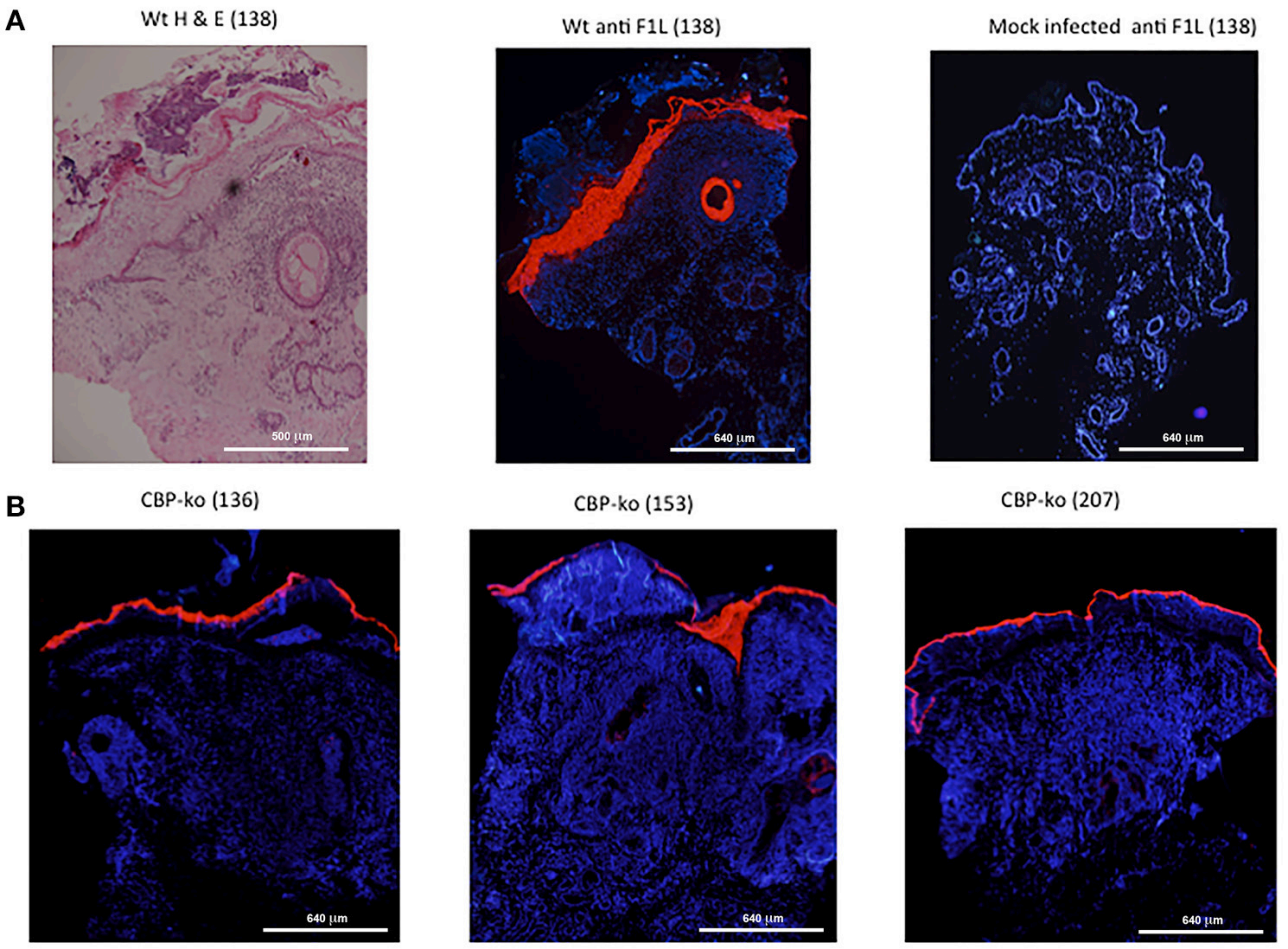
**Histological sections from punch biopsies of virus-infected animals were stained by H & E or by immunohistochemical staining for the ORFV envelope protein F1L. (A)** Day 4 p.i. at a dose of 10^7^ p.f.u. *wt* virus. Adjacent sections were stained by H & E and ORFV F1L antigen. In addition ant-FIL staining was performed for the mock-infected (PBS) control. Staining for ORFV infected tissue is seen localized to the epidermis and in epidermal cells of the hair follicle. **(B)** Typical F1L staining in animals infected with the CBP knock-out virus at a dose of 10^7^ p.f.u. virus at day 6 p.i. The biopsies were taken from papules/pustules that had developed along the virus-inoculated scratch line. The images were taken at 4X magnification.

In order to establish whether or not the CBP-knock-out virus replicated in sheep-skin, biopsy tissue sections were stained for the ORFV F1L major immunodominant antigen that is associated with the virus envelope. F1L antigen staining is clearly seen within the epidermal layer for the *wt* virus (day 4 p.i.) where contiguous sections were stained by H & E and for F1L (Figure 7A). There was no staining detected for the mock-infected control (Figure 7A). In the case of the CBP-knock-out virus, biopsies were taken where there was evidence of a raised papule or pustule. F1L antigen staining clearly showed that the CBP-knock-out virus established productive infection in all animals within this group. Figure 7B shows examples of typical staining for 3 of the animals in the CBP ko virus at day 6 p.i.

### Histology analysis of CBP-knock virus-infected skin tissue revealed changes typical of the *wt* virus

**Figure 8:**
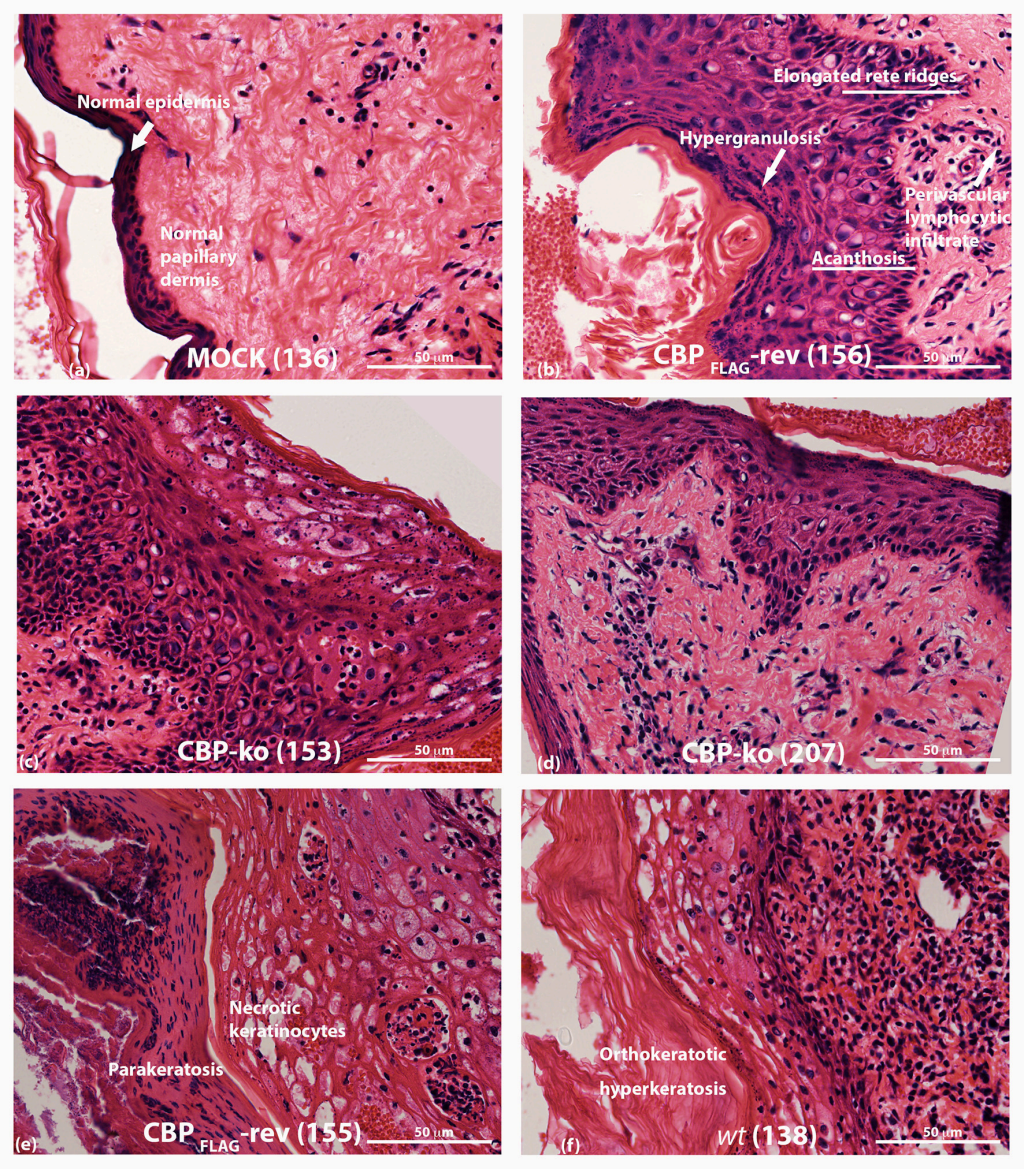
**Histopathology of ORFV infected skin tissue**. H & E stained sections from biopsy tissue at day 4 p.i., dose 10^7^ of p.f.u. virus. **(A)** mock-infected (PBS) skin **(B)** CBP_*FLAG*_ -revertant showing histological features of infected tissue **(C,D)** histological features of CBP-knockout virus **(E)** CBP-revertant virus infected tissue showing parakeratosis and necrotic keratinocytes **(F)** *wt* infected tissue showing hyperkeratosis. The images were taken at 40X magnification.

ORFV infected tissue was analyzed by H & E staining at day 4 p.i. As described above, biopsies for the CBP-ko group were taken from the skin where there was evidence of a raised papule or pustule. Animals infected with the CBP-ko virus showed marked acanthosis (thickening of the stratum basal and stratum spinosum), elongated rete ridges and orthokeratotic hyperkeratosis (thickening of stratum corneum) typical of the *wt* virus (Figure 8). The same histological features were also seen for the revertant viruses. This was not seen for the mock-infected control group. All infected animals had increased (moderate to extreme) inflammatory infiltrate, most notable within the papillary dermis, but also entering the epidermis. In all virus-infected groups hypergranulosis (thickened cornified layer) and parakeratosis (cell nuclei present in the cornified layer) were present (Figure 8). Again these features were not seen in the mock-infected group.

The H & E staining revealed that the inflammatory cell infiltrate was similar but variable for *wt*, CBP-ko and CBP revertant viruses during the early stages of infection (day 4) when clinical signs of infection were first becoming apparent. At a dose of 10^7^ virus per inoculum, the numbers of inflammatory cells for the *wt*, CBP-ko and CBP revertants varied from medium to heavy. At a dose of 10^5^ virus per inoculum, there was a similar influx of inflammatory cells but again this was variable for infected animals within groups. In the PBS controls there were few inflammatory cells seen (Figure 8A).

### MHC-II staining revealed differences between the virus-infected groups

**Figure 9:**
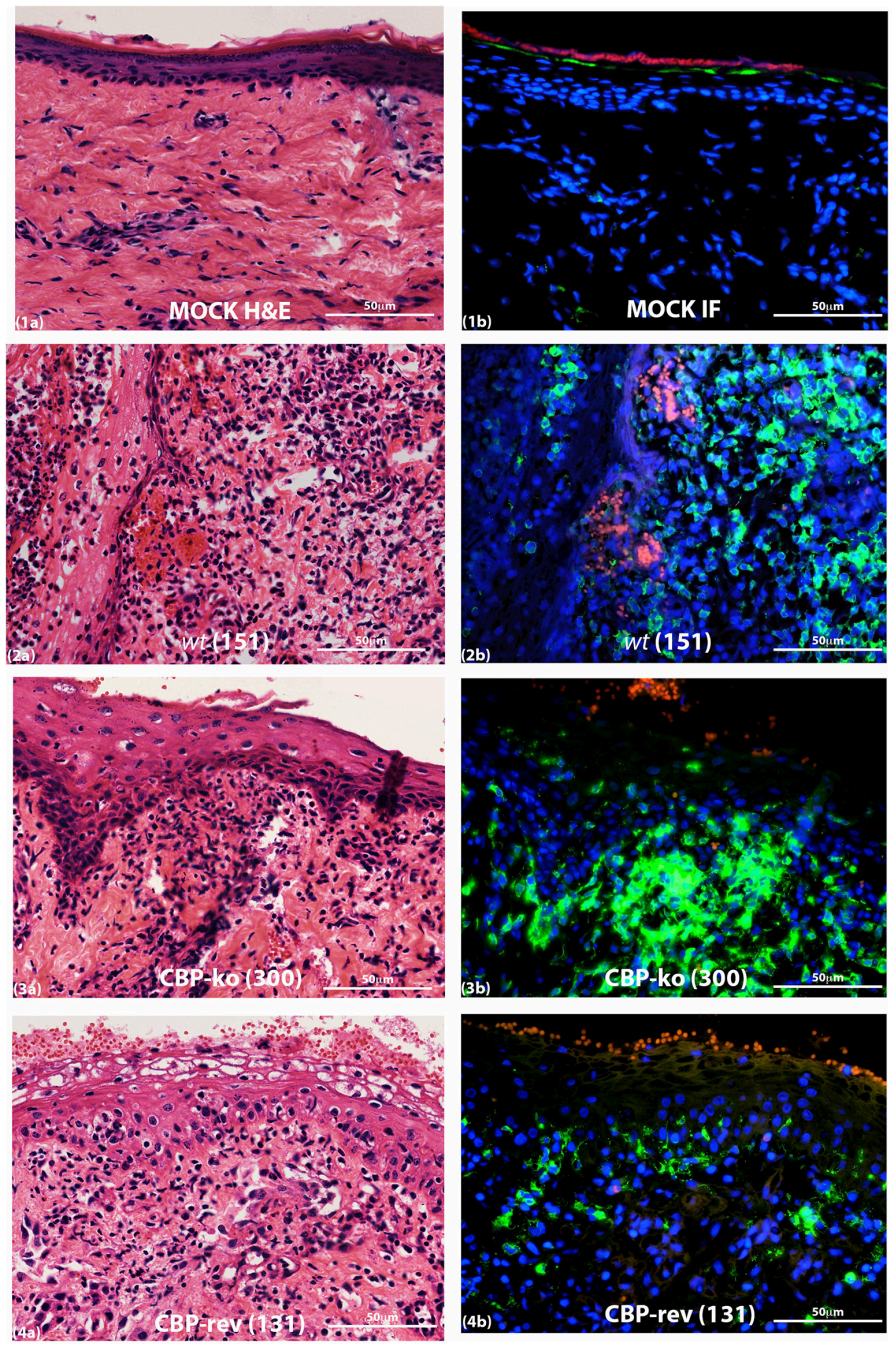
**MHC-II staining of ORFV infected skin tissue and mock infected**. Biopsy tissue day 4 p.i., dose 10^7^ p.f.u. virus. Adjacent sections for H & E **(a)** and MHC-II staining **(b)** are shown: **(1)** mock infected (PBS), **(2)** *wt*, **(3)** CBP-knock-out virus, **(4)** CBP revertant virus. MHC-II positive cells (green); cell nuclei (DAPI) (blue); non-specific staining of red blood cells (red). The images were taken at 40X magnification.

MHC-II staining at day 4 p.i. is shown in Figure 9. The adjacent sections show that for mock-infected (PBS) tissue there was very little MHC-II staining either within the papillary dermis or within the epidermis. For virus-infected tissue however MHC-II staining increased for all the groups but was variable for virus-infected animals within groups. All mock infected skin tissue showed clearly defined and intact nuclei in the dermis by DAPI staining, however virus infected animals consistently showed histopathological changes in the nuclei that were not seen in the mock infected tissue, that is the edges of nuclei for virus infected skin were not as sharply defined and appeared smeared compared with mock infected skin tissue (Figure S4).

**Figure 10:**
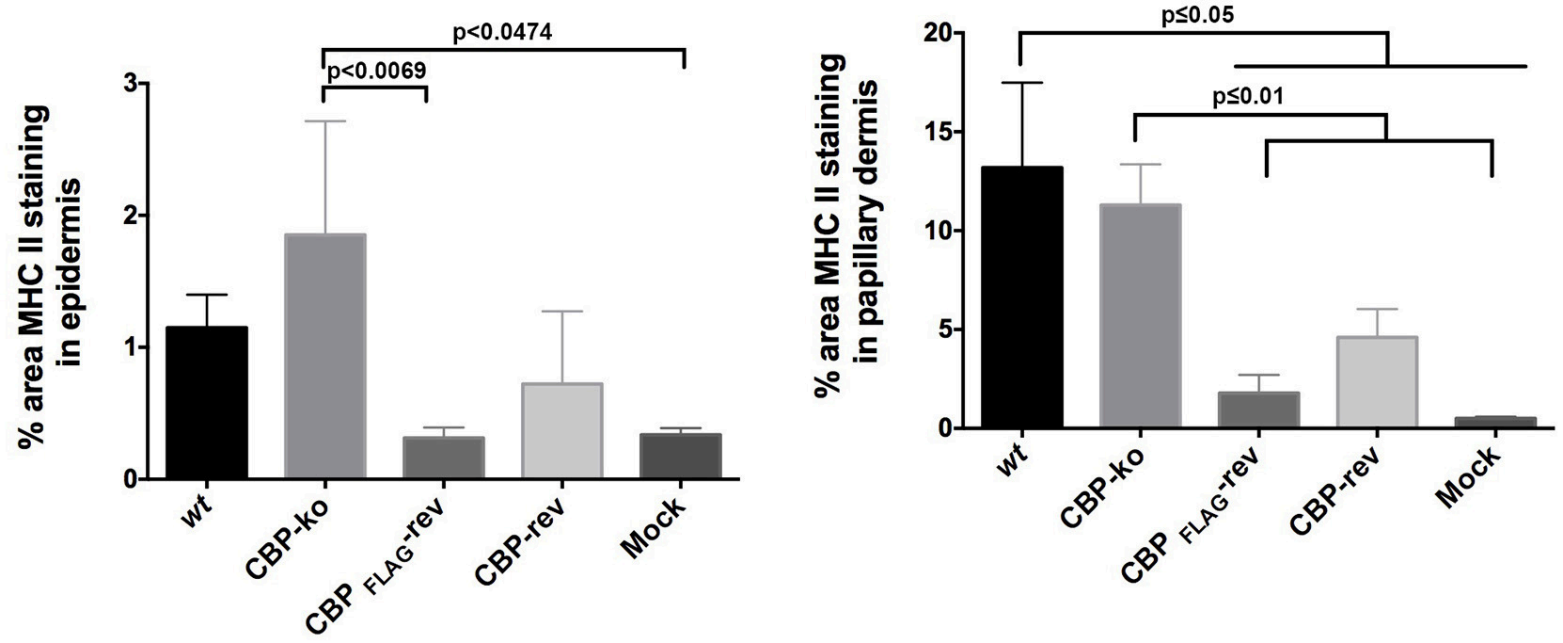
**MHC-II staining in the epidermis and papillary dermis**. The percent area for MHC-II staining was determined using Image J Fiji. Biopsy tissue day 4 p.i., dose 10^7^ p.f.u. virus and for mock infected (PBS). The data was analyzed by One Way ANOVA plus Sidoks test where *N* = 6 for *wt*, CBP-knockout (KO), CBP-FLAG revertant, and CBP revertant, that is one biopsy taken from each infected animal. *N* = 22 for mock infected (biopsies taken from animals across all 4 groups).

The quantification of MHC-II staining is shown in Figure 10. The percent area MHC-II staining in the epidermis for the CBP-ko virus group was higher compared with the *wt* virus group and animals infected with the revertant viruses, however the percent area staining was only significantly higher for the CBP-ko virus compared with the CBP-_FLAG_-rev virus (*P* = 0.0069). Interestingly the *wt* and both revertant viruses were not significantly different to the mock-infected animals whereas the CBP-knock-out-virus was significantly higher than the mock-infected control group (*P* = 0.0479). The percent area MHC-II staining in the papillary dermis revealed slightly higher staining for MHC-II for the *wt* virus compared with the CBP-ko virus, however the difference was not significant. Interestingly the percent area staining of MHC-II cell in the papillary dermis was significantly less for both revertant viruses compared with the CBP-ko virus and the *wt* virus (*P* < 0.01 and *P* < 0.05 respectively). The percent area MHC-II staining in the papillary dermis for the revertant viruses was again not significantly different to the mock-infected animals.

### A primary antibody response was detected against the ORFV 075 scaffold protein in sheep infected with the CBP knock-out virus

**Figure 11:**
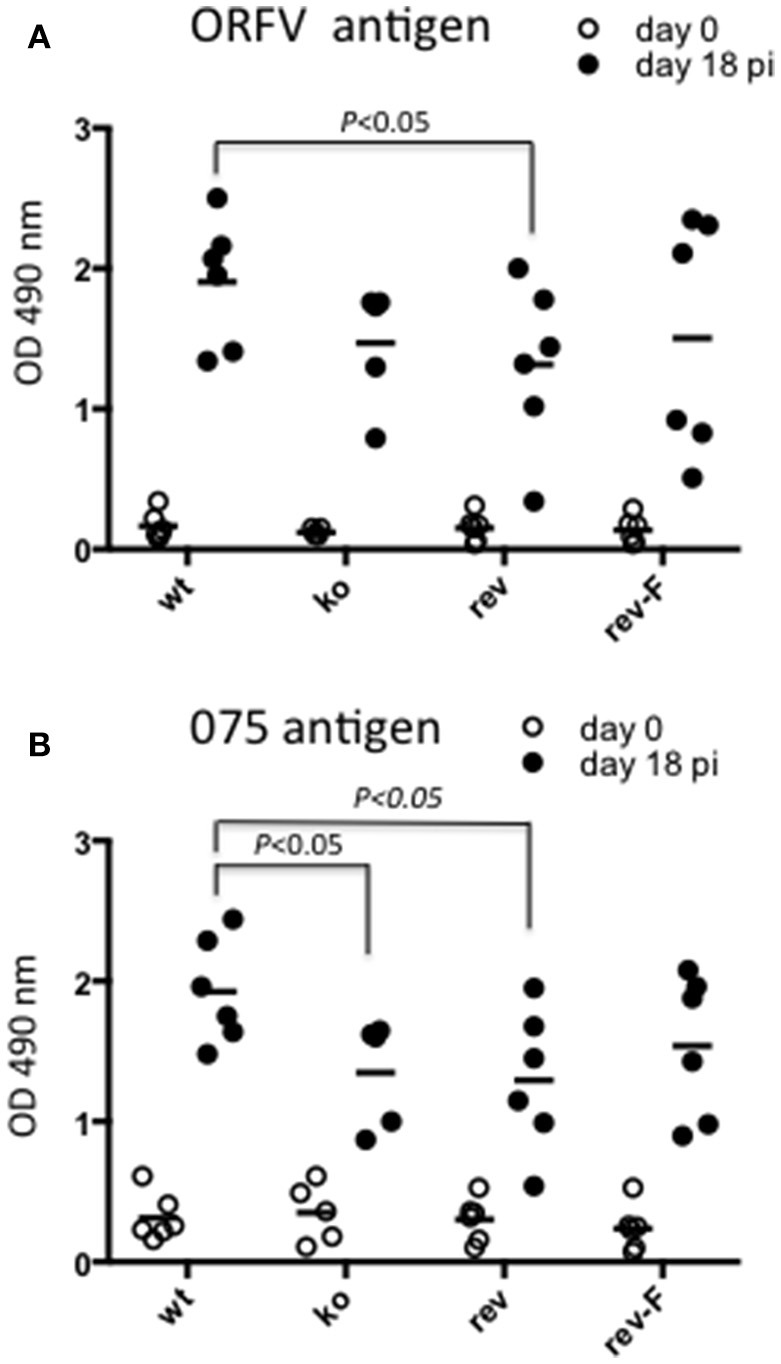
**Serological analysis of anti-ORFV antibodies in infected sheep**. Sheep sera from animals infected with either ORFV *wt* or CBP-knock-out virus (ko) or CBP-revertant virus (rev) or CBP_FLAG_ revertant (rev-F) were tested for antibodies against ORFV antigen **(A)** or the ORFV scaffold protein ORFV075 **(B)** at 0 and 18 days p.i. by ELISA. Shown are the serum antibody absorbance values for each animal (serum dilution 1/400) and the mean serum antibody absorbance values for each group. *P* values determined by Mann-Whitney test.

We were interested to determine whether animals produced an immune response against antigen produced by the virus in infected cells by *de novo* synthesis. ORFV-075 is a scaffold protein that is produced late in infection but is not incorporated into the viral particle. Figure 11A shows that all animals had seroconverted and produced a typical antibody response against ORFV antigen by day 18 pi. There was no significant difference in antibody levels between the *wt* and CBP ko virus (*P* = 0.061) however there was a significant difference between the *wt* and the revertant (*P* = 0.0465). Detection of antibodies against ORFV-075 showed that the ORFV-CBP-knock-out virus had replicated in all infected animals (Figure 11B). A significant difference was found between the *wt* and the CBP ko virus in this case (*P* = 0.0274) with the *wt* producing a higher antibody response. Further there was a significant difference in antibody levels for the *wt* compared with the revertant virus (*P* = 0.0465) but not between the other groups.

## Discussion

We firstly characterized the biological properties of the purified ORFV-NZ7 CBP protein and then investigated the effects of deleting the CBP gene from ORFV on replication, virulence, pathogenesis, and host response. As described above, the CBP gene shows surprising variability within the ORFV species with the NZ7 strain showing 78% identity to the NZ2 strain (Seet et al., 2003). Because of these genetic differences, experiments were conducted initially to investigate and characterize the biological properties of the putative CBP_NZ7_ prior to investigations in sheep with recombinant virus. Chemokine binding assays showed that it displayed the equivalent biological properties as CBP_NZ2_ despite differences in their primary structure. In addition, CBP_NZ7_ potently blocked the inflammatory cell infiltrate when injected intradermally at ng levels in a mouse skin model, suggesting that it could have a role in pathogenesis.

In the context of ORFV infection of sheep, the deletion of the CBP gene had a marked effect on the clinical pathology. Within the first 3 days many typical small papules formed in the CBP knock-out group, however, surprisingly few of these papules progressed further to form pustules and in fact diminished with time. There was only one sheep infected with the CBP-knockout virus that showed signs of pustular development by day 6 p.i. There was little evidence in the case of the other animals within this group that the CBP-knockout virus had established infection. To demonstrate replication, we were able to detect viral antigen in tissue by immunofluorescence from biopsies specifically taken from the few raised papules that were observed in each animal within this group. Within the papules the histology of the CBP-knockout virus infected tissue resembled the *wt* and revertant viruses with evidence of acanthosis, elongated rete ridges and hyperkeratosis. Furthermore by day 4 p.i., it was evident that there was a medium to large inflammatory cell infiltrate in the *wt*, CBP-knock-out virus and CBP-revertant virus lesions by H & E staining.

We investigated the infiltration of immune cells into the infected site by staining for MHC-II. The percent area staining for MHC-II suggested that there was greater infiltration of the papillary dermis in the case of the CBP-knock out virus group compared with the revertant virus groups but not compared with the *wt* group. In addition there was significantly higher MHC-II staining in the epidermis for the CBP-knock-out virus compared with the CBP-FLAG revertant virus group. The results suggest, for the recombinant viruses at least, that secreted CBP from virus infected cells in the epidermis maybe disrupting chemokine gradients and reducing immune cells reaching the site of infection. Its puzzling as to the reason for the higher percent area staining for MHC-II for the *wt* compared with the revertant viruses, both in the epidermis and papillary dermis, when the clinical lesions appeared similar. If the secreted CBP is preventing immune cells reaching the site of infection an inverse relationship might be predicted between MHC-II staining and the severity of the clinical lesions. It's possible that within the first few days post-infection, the critical cellular immune events that determine the establishment of infection have occurred. Perhaps biopsy material taken from the infected area before signs of viral pathology emerged might have provided insight into such immune events.

Humoral antibody analysis provided a measure of the induction of the adaptive responses. The anti-ORFV antigen levels for the CBP knock-out virus were comparable with the *wt* virus and the revertants although there were few outward signs of clinical infection in the CBP-knockout virus at the highest dose, apart from papules within the first 3–5 days. There appeared to be little correlation between the magnitude of clinical lesions over the course of the experiment and the serum antibody levels detected at 18 days p.i. It's possible that the adaptive responses could have been mobilized more rapidly in the case of the CBP-knock-out virus where immature DC trafficking to the skin and migration of mature DC to peripheral lymph nodes would have been less disrupted than for the intact virus. The humoral antibody response normally takes about 4–5 days to develop (Janeway et al., 1999) and we did not determine whether this response was initiated more rapidly in animals infected with the CBP-knock-out virus compared with the *wt* or CBP-revertants.

The only other study in which a poxvirus CBP knock-out virus has been tested in the context of *in vivo* infection is rabbitpox virus (RPV) (Graham et al., 1997). In that case the virus was injected intradermally. The CBP knock-out RPV showed an influx of at least two classes of leukocytes at 3 days post-infection. Influx of CD43-positive lymphocytes (possibly NK cells), monocyte/macrophages as well as CD43-negative granulocytes in the knock-out virus. The results suggested that the expression of the RPV 35 kDa secreted CBP functions during the early stages of infection to reduce the initial influx of extravasating leukocytes. Although we suspect that the ORFV CBP most likely reduced the influx of such cell types identified in RPV infections, we were not able to establish whether or not the ORFV CBP influenced leukocyte migration. Overt signs of ORFV clinical infection are generally not apparent until 3 days p.i. and it's possible that the effects of the CBP gene were manifested before this time.

Our studies clearly showed that the CBP gene is critical in ORFV pathogenesis and virulence and although ORFV encodes a number of other secreted anti-inflammatory factors, the deletion of CBP gene severely attenuated the virus. The secreted CBP appears to induce its effects early during infection, since there were few signs of clinical orf in almost all CBP knock-out virus infected animals. At this time we do not know which cells or factors were involved in the faster control of the ORFV CBP-knock-out virus infection. The deletion of the CBP gene may however be advantageous for recombinant ORFV vaccine development. Although its deletion severely attenuated the virus, a primary antibody response typical of the *wt* virus was observed.

## Author contributions

SF, ZL, and LW designed and conceived the experiments. SF, NR, CM, ZL, and AD performed the experiments. SF wrote the paper. LW and AM assisted with the manuscript preparation and proof reading.

### Conflict of interest statement

The authors declare that the research was conducted in the absence of any commercial or financial relationships that could be construed as a potential conflict of interest.

## Supplementary material

Figure S1 **Growth of recombinant ORFVs in cell culture**. Lamb testis cells at 5 × 10^5^ were infected with either *wt*, ORFV-CBP knock-out (CBP-ko), ORFV CBP revertant (CBP-rev) or ORFV CBP_FLAG_ revertant (CBP_FLAG_-rev) at an MOI of 0.2, 1 and 5 p.f.u/cell in triplicate. At 30 h p.i. virus was harvested and the virus levels determined by plaque assay (Balassu and Robinson, 1987). Mean ± SD is shown.

Figure S2 **Northern blot analysis of total RNA isolated from LT cells infected with recombinant ORFV**. Cells were infected with ORFV in the presence of cycloheximide (100 μg/ml) at an MOI of 30 pfu. RNA was isolated at 6 h post-infection. Hybridisation was performed with a CBP-specific ^32^P-labeled DNA probe. Bands indicate mRNA of 900–1000 bp. Cells were either mock infected or infected with ORFV-CBP-_FLAG_-revertant,ORFV-CBP-revertant ORFV-CBP-knockout and ORFV wild type.

Figure S3 **Western blot analysis of CBPexpression by CBP_**FLAG**_-revertant ORFV**. LT cells were infected with virus and incubated for 5 days. Both the supernatant and cell lysate were collected for analysis of CBP_FLAG_ protein. Protein was enriched by affinity chromatography using α-FLAG-M2 Affinity Gel. Proteins were detected with anti-FLAG antibody. Lane 1 mock infected, lane 2 CBP_FLAG_ purified protein, lane 3 ORFV-CBP_FLAG_-revertant (cell lysate), lane 4 ORFV-CBP-revertant (supernatant), lane 5 ORFV-CBP_FLAG_ (supernatant).

Figure S4 **Comparison of DAPI staining in biopsy tissue from sheep either mock-infected or infected with ORFV CBP-ko at a dose of 10^**7**^ pfu at day 4 p.i**.
