# Supplementary figures and images for: Deletion of the Chemokine Binding Protein Gene from the Parapoxvirus Orf Virus Reduces Virulence and Pathogenesis in Sheep
Source: Front Microbiol. 2017 Jan 24;8:46. doi: 10.3389/fmicb.2017.00046 (PMC5258736; doi:10.3389/fmicb.2017.00046)

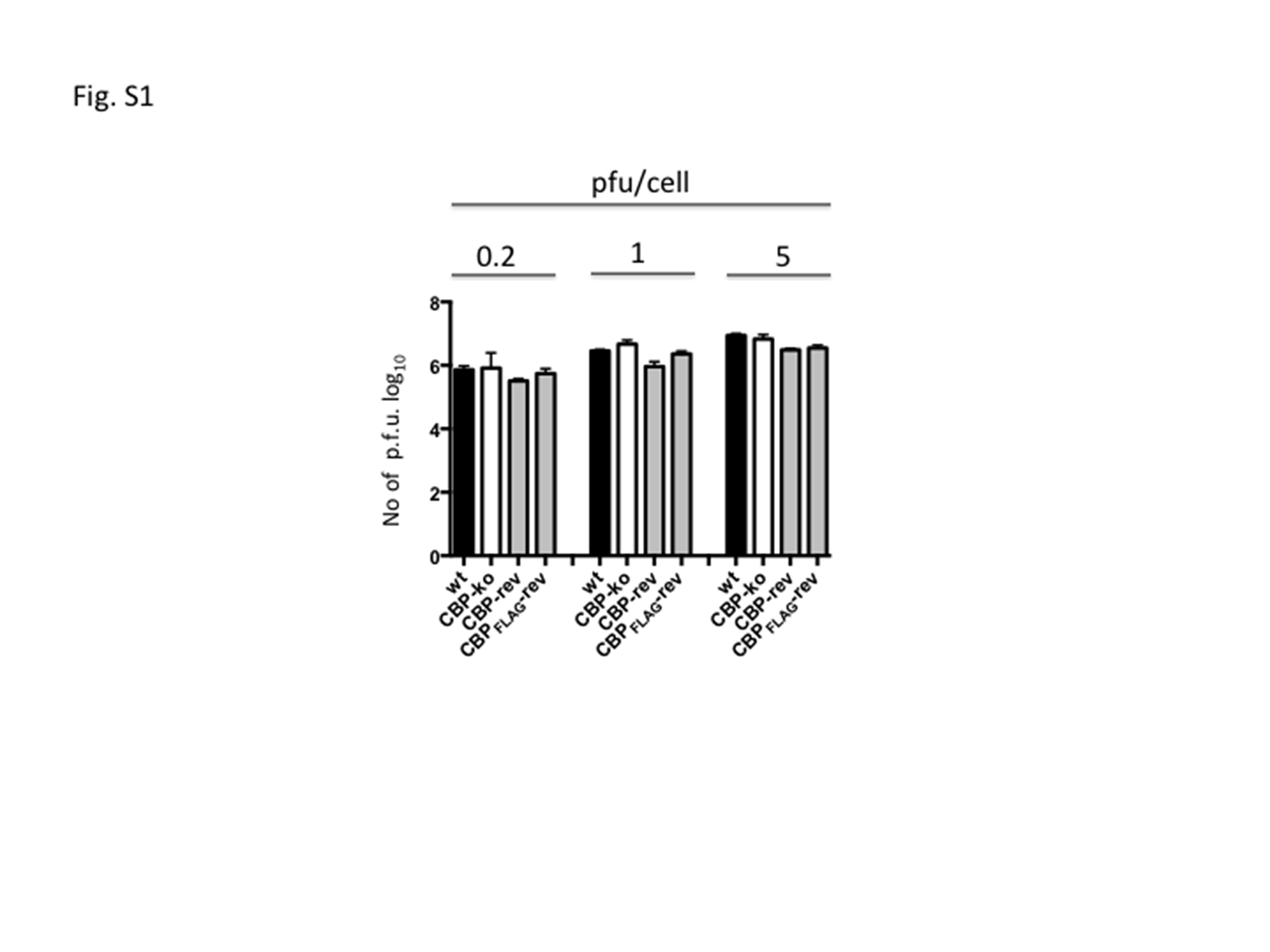

Supplement: Figure S1 — Growth of recombinant ORFVs in cell culture. Lamb testis cells at 5 × 10^5^ were infected with either wt, ORFV-CBP knock-out (CBP-ko), ORFV CBP revertant (CBP-rev) or ORFV CBPFLAG revertant (CBPFLAG-rev) at an MOI of 0.2, 1 and 5 p.f.u/cell in triplicate. At 30 h p.i. virus was harvested and the virus levels determined by plaque assay (Balassu and Robinson, 1987). Mean ± SD is shown. [file Image1.TIFF]

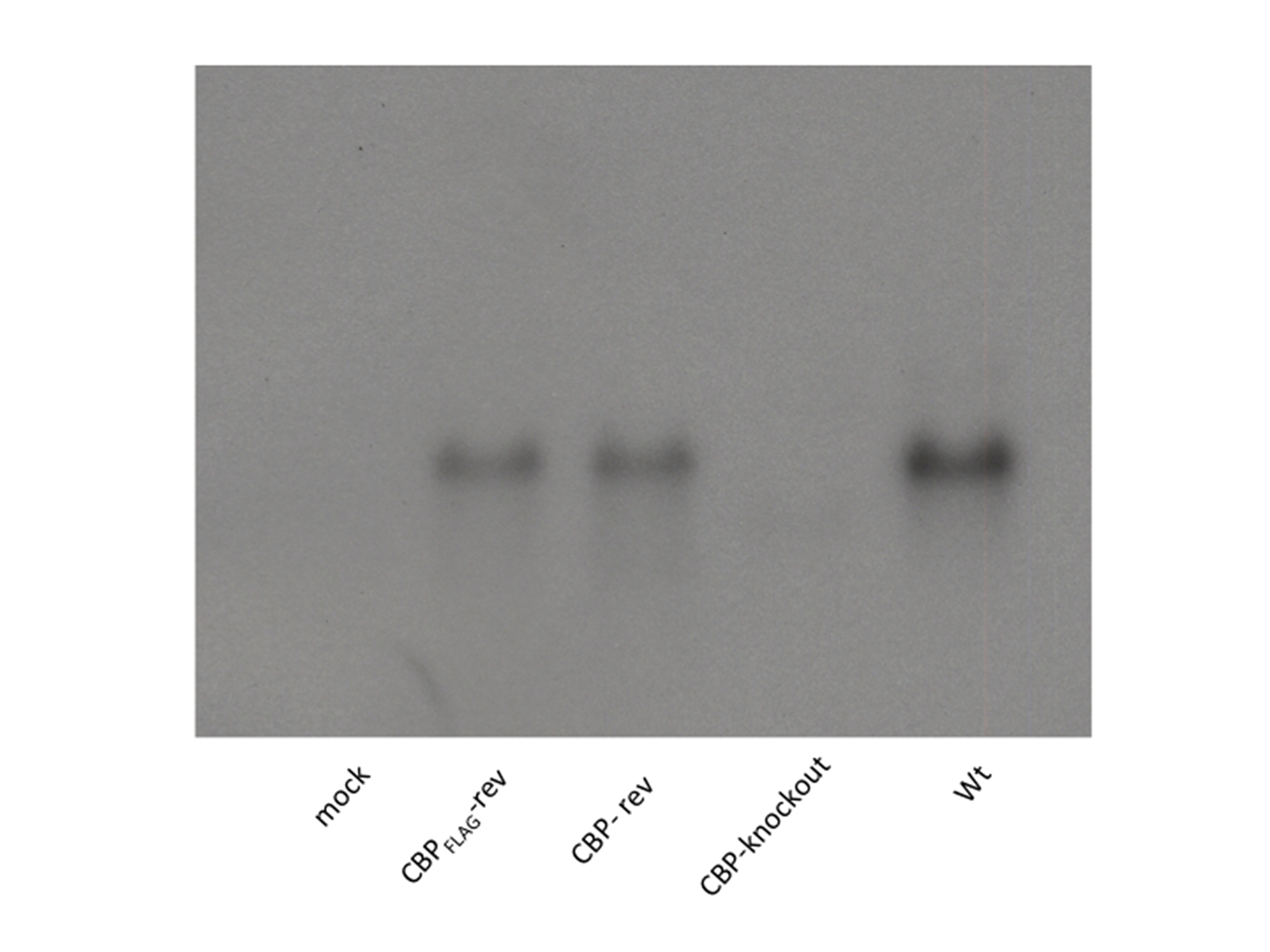

Supplement: Figure S2 — Northern blot analysis of total RNA isolated from LT cells infected with recombinant ORFV. Cells were infected with ORFV in the presence of cycloheximide (100 μg/ml) at an MOI of 30 pfu. RNA was isolated at 6 h post-infection. Hybridisation was performed with a CBP-specific 32P-labeled DNA probe. Bands indicate mRNA of 900–1000 bp. Cells were either mock infected or infected with ORFV-CBP-FLAG-revertant,ORFV-CBP-revertant ORFV-CBP-knockout and ORFV wild type. [file Image2.TIFF]

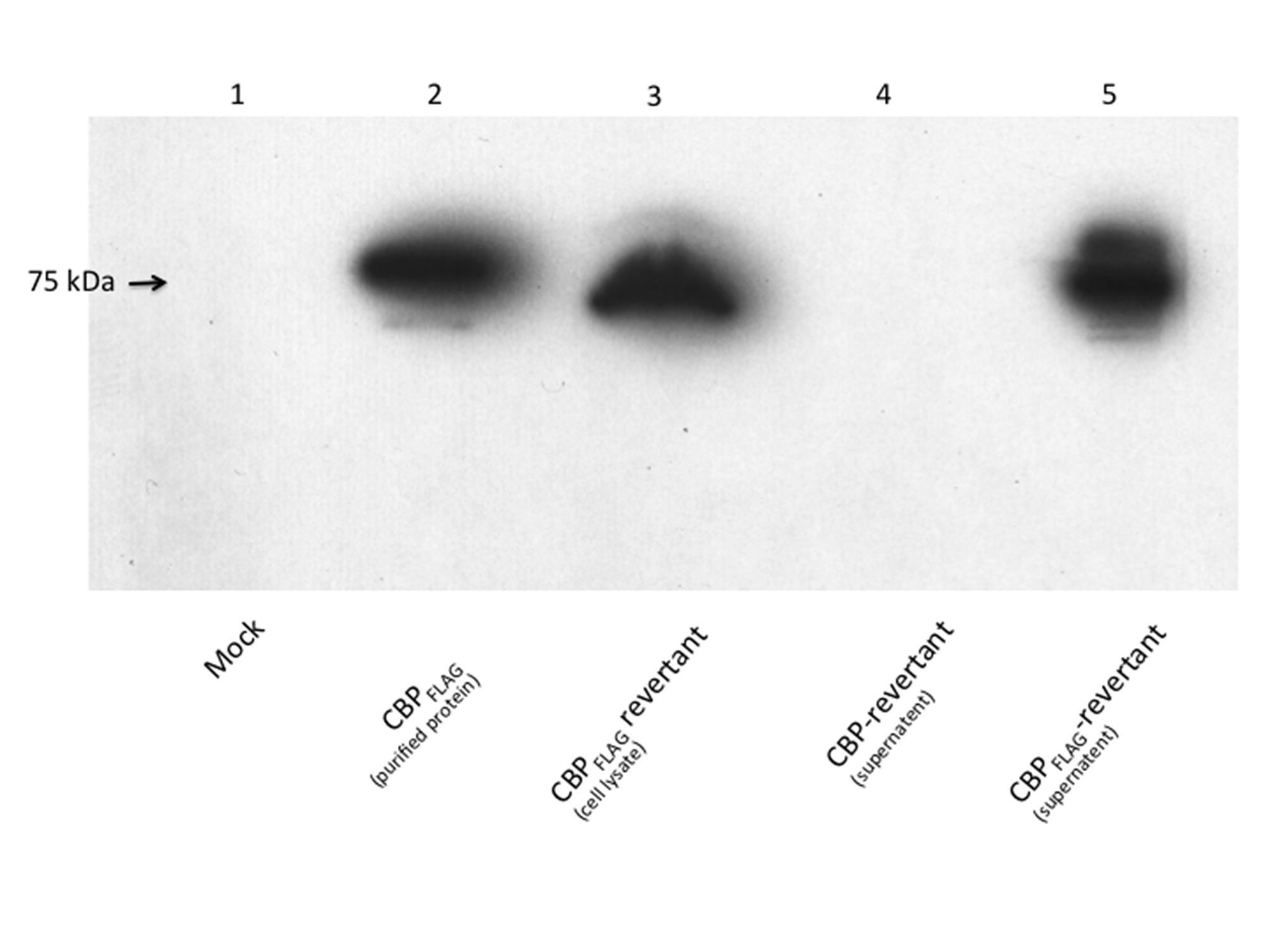

Supplement: Figure S3 — Western blot analysis of CBPexpression by CBPFLAG-revertant ORFV. LT cells were infected with virus and incubated for 5 days. Both the supernatant and cell lysate were collected for analysis of CBPFLAG protein. Protein was enriched by affinity chromatography using α-FLAG-M2 Affinity Gel. Proteins were detected with anti-FLAG antibody. Lane 1 mock infected, lane 2 CBPFLAG purified protein, lane 3 ORFV-CBPFLAG-revertant (cell lysate), lane 4 ORFV-CBP-revertant (supernatant), lane 5 ORFV-CBPFLAG (supernatant). [file Image3.TIFF]

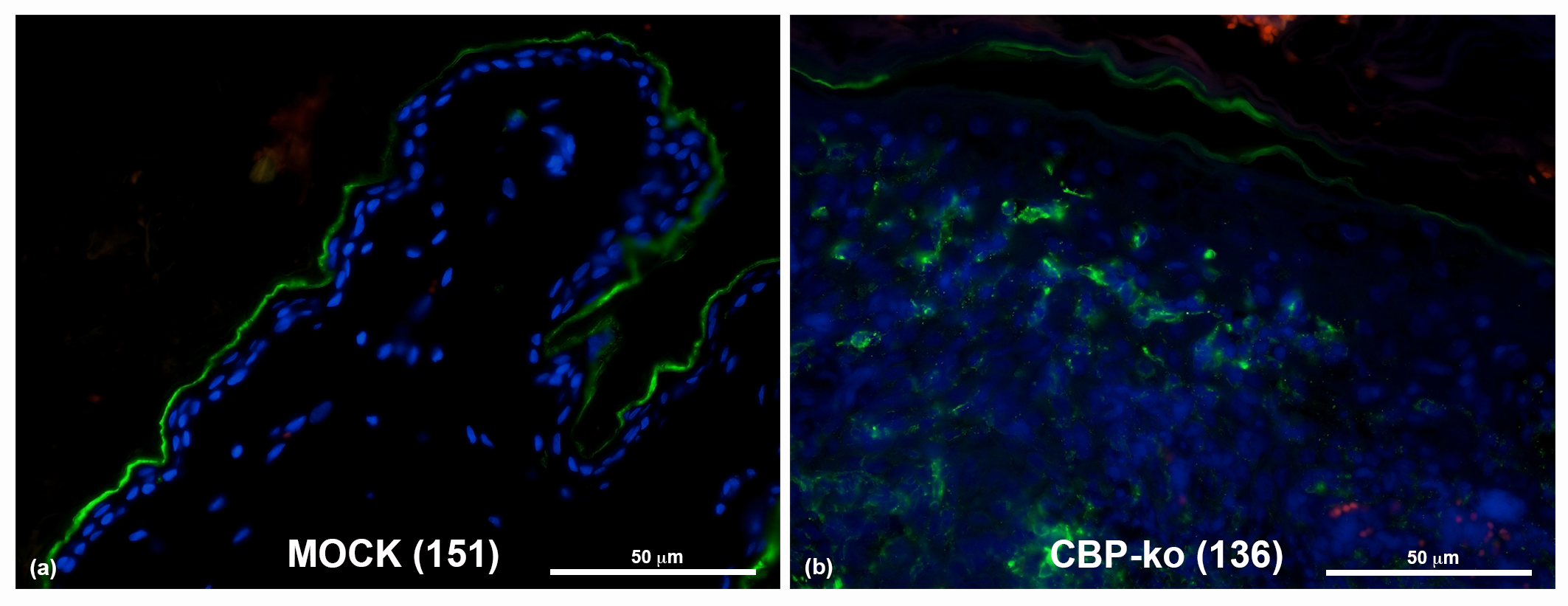

Supplement: Figure S4 — Comparison of DAPI staining in biopsy tissue from sheep either mock-infected or infected with ORFV CBP-ko at a dose of 107 pfu at day 4 p.i. [file Image4.TIF]
